# Personalized objects can optimize the diagnosis of EMCS in the assessment of functional object use in the CRS-R: a double blind, randomized clinical trial

**DOI:** 10.1186/s12883-018-1040-5

**Published:** 2018-04-12

**Authors:** Yuxiao Sun, Jianan Wang, Lizette Heine, Wangshan Huang, Jing Wang, Nantu Hu, Xiaohua Hu, Xiaohui Fang, Supeng Huang, Steven Laureys, Haibo Di

**Affiliations:** 1 International Vegetative State and Consciousness Science Institute, Hangzhou Normal University, Hangzhou, China; 2 GIGA, GIGA-Consciousness, Coma Science Group, University & Neurology Department, Hospital of Liege, Liege, Belgium; 3 Audition Cognition and Psychoacoustics Team, Lyon Neuroscience Research Center, Lyon, France; 4 Rehabilitation Center for Brain Damage, Wujing Hospital of Hangzhou City, Hangzhou, China

**Keywords:** Coma recovery scale-revised, Functional object use, Personalized objects, Non-personalized objects

## Abstract

**Background:**

Behavioral assessment has been acted as the gold standard for the diagnosis of disorders of consciousness (DOC) patients. The item “Functional Object Use” in the motor function sub-scale in the Coma Recovery Scale-Revised (CRS-R) is a key item in differentiating between minimally conscious state (MCS) and emergence from MCS (EMCS). However, previous studies suggested that certain specific stimuli, especially something self-relevant can affect DOC patients’ scores of behavioral assessment scale. So, we attempted to find out if personalized objects can improve the diagnosis of EMCS in the assessment of Functional Object Use by comparing the use of patients’ favorite objects and other common objects in MCS patients.

**Methods:**

Twenty-one post-comatose patients diagnosed as MCS were prospectively included. The item “Functional Object Use” was assessed by using personalized objects (e.g., cigarette, paper) and non-personalized objects, which were presented in a random order. The rest assessments were performed following the standard protocol of the CRS-R. The differences between functional uses of the two types of objects were analyzed by the McNemar test.

**Results:**

The incidence of Functional Object Use was significantly higher using personalized objects than non-personalized objects in the CRS-R. Five out of the 21 MCS studied patients, who were assessed with non-personalized objects, were re-diagnosed as EMCS with personalized objects (χ^2^ = 5, df = 1, *p* < 0.05).

**Conclusions:**

Personalized objects employed here seem to be more effective to elicit patients’ responses as compared to non-personalized objects during the assessment of Functional Object Use in DOC patients.

**Trial registration:**

Clinical Trials.gov: NCT02988206; Date of registration: 2016/12/12.

**Electronic supplementary material:**

The online version of this article (10.1186/s12883-018-1040-5) contains supplementary material, which is available to authorized users.

## Background

Behavioral assessment is the gold standard for diagnosis in patients with disorders of consciousness (DOC) [1, 2], and the Coma Recovery Scale-Revised (CRS-R) is an important tool for the assessment of DOC such as the “vegetative state” (now also coined unresponsive wakefulness syndrome; VS/UWS) [3] and the minimally conscious state (MCS) [4]. Differential diagnosis of the level of consciousness in patients with DOC is of great importance, for instance in the decisions on treatment, care, and end-of-life [5]. Clinical diagnosis is based on reliable behavioral responsiveness to the stimuli suggested by the CRS-R. The lower scores for each sub-scale represent reflexive behavior and the higher scores represent relatively conscious behavior. Several items from different sub-scales identify consciously mediated behaviors, and final diagnosis is based on the best behavior over all sub-scales (and multiple assessments).

When the patient shows Functional Object Use (score 6 in the motor function sub-scale) or Accurate Functional Communication (score 2 in the communication sub-scale), he/she is diagnosed as an EMCS, which is no longer a DOC. Difference between EMCS and MCS coincide with an increase in functional connectivity collaboration between anti-correlated brain networks [6]. The difference between MCS and EMCS can clinically be based on responses to presented stimuli (e.g., Functional Object Use). Previous research has shown that self-referential stimuli are more effective to elicit patient’s responses than non-self-referential stimuli [5, 7]. Laureys et al. found that patients with MCS had a broader range of brain activation using their own names than using any other sound stimuli when patients were scaned with PET [8]. Perrin et al. found that preserved semantic processing could be observed in non-communicative brain-damaged patients, particularly for the subject’s own name [9]. In addition, a study with functional magnetic resonance imaging (fMRI) found that 7 patients with VS/UWS who responded to their own names became MCS after 3 months and showed the value of using patient’s own name [10]. Other self-referential stimuli such as familiar faces result in a higher number of responses [11]; a preferred music (i.e., a self-referential, autobiographical and emotional stimulus) [12], or a familiar voice of a relative has an effect on behavioral and cognitive processes of patients with DOC [12].

It is thus hypothesized that self-referential stimuli might improve diagnostic assessment, and subsequently decrease the level of misdiagnosis.

To our knowledge, different stimuli indeed have different effects on the behavioral response of patients. The aim of the present study is to compare the frequency of Functional Object Use elicited by the stimuli suggested by the CRS-Rand the personalized stimuli in order to optimize the behavioral assessment of the motor function sub-scale.

## Methods

Twenty-one patients diagnosed as MCS were recruited (standard diagnosis procedure using the CRS-R for 4 times within 2 weeks; 11 patients were diagnosed as MCS+ and 10 patients as MCS-; mean age 54.67 ± 13.58 years old; 15 male, 6 female); mean time since injury was 6 ± 3.81 months). Each patient had the motor ability to move their hands and arms (i.e., motor sub-scale scores ≥1 by using noxious stimuli to upper limbs) [13]. Etiology was traumatic in 11 (52%) patients and non-traumatic in 10 (48%) patients. Table 1 shows the clinical data for each of the 21 patients diagnosed as MCS using the CRS-R. The study was approved by the Ethics Committee of Hangzhou Normal University and Wujing Hospital which complies with the Code of Ethics of the World Medical Association (Declaration of Helsinki). Written informed consents were obtained by the patient’s legal surrogates.

**Table 1:** Function Object Use with personalized objects

Patient	Aetiology /Time since injury (month)	CRS-R^1^			CRS-R Personalized Objects		
		Diagnosis	Functional Object Use^2^		Re-Diagnosis	Functional Object Use	
			comb	cup		Object 1	Object 2
1	Traumatic/8	MCS-(2-1-3-2-0-3)	0/2	0/2	EMCS (2-2-6-1-0-3)	Paper (2/2)	Pen (2/2)
2	Non-traumatic/6	MCS+(2-1-2-2-1-2)	0/2	0/2	MCS+(2-1-2-2-1-2)	0/2	0/2
3	Non-traumatic/3	MCS+(3-5-3-2-1-3)	0/2	0/2	EMCS (3-5-6-2-1-3)	Paper (2/2)	Pen (2/2)
4	Traumatic/7	MCS+(2-1-2-2-1-2)	0/2	0/2	MCS+(2-1-2-2-1-2)	0/2	0/2
5	Traumatic/3	MCS+(1-3-3-2-0-2)	0/2	0/2	MCS+(3-3-3-3-1-2)	0/2	0/2
6	Non-traumatic/5	MCS+(3-1-4-1-0-2)	0/2	0/2	MCS+(3-1-4-1-0-2)	0/2	0/2
7	Traumatic/10	MCS-(0-2-2-1-0-2)	0/2	0/2	MCS-(0-2-2-1-0-2)	0/2	0/2
8	Traumatic/3	MCS-(1-3-1-1-0-2)	0/2	0/2	EMCS (1-3-6-1-0-2)	Phone (2/2)	Tooth Brush (2/2)
9	Non-traumatic/18	MCS-(2-1-2-1-1-2)	2/2	0/2	EMCS (1-2-6-1-0-3)	Comb (2/2)	Phone (2/2)
10	Non-traumatic/10	MCS-(2-0-1-2-0-2)	0/2	0/2	EMCS (3-5-6-2-1-3)	Phone (2/2)	Fan (2/2)
11	Non-traumatic/6	MCS+(3-5-1-2-1-2)	0/2	0/2	MCS+(3-5-1-2-1-2)	0/2	0/2
12	Traumatic/6	MCS+(2-3-2-2-1-2)	0/2	0/2	MCS+(2-2-2-2-1-2)	0/2	0/2
13	Non-traumatic/3	MCS+(4-5-2-3-1-3)	0/2	0/2	MCS+(4-5-2-3-1-3)	0/2	0/2
14	Traumatic/3	MCS+(4-5-4-2-1-2)	0/2	0/2	MCS+(4-5-4-2-1-2)	0/2	0/2
15	Non-traumatic/2	MCS+(4-5-4-2-0-2)	0/2	0/2	MCS+(4-5-4-2-0-2)	0/2	0/2
16	Non-traumatic/5	MCS-(2-3-3-1-0-2)	0/2	0/2	MCS-(2-3-3-1-0-2)	0/2	0/2
17	Traumatic/9	MCS-(2-3-2-1-0-2)	0/2	0/2	MCS-(2-3-2-1-0-2)	0/2	0/2
18	Traumatic/1	MCS-(1-1-4-1-0-2)	0/2	0/2	MCS-(2-1-4-1-0-2)	0/2	0/2
19	Non-traumatic/9	MCS+(4-5-2-2-0-2)	0/2	0/2	MCS+(4-5-2-2-0-2)	0/2	0/2
20	Traumatic/4	MCS-(2-3-3-1-0-2)	0/2	0/2	MCS-(2-3-3-1-0-2)	0/2	0/2
21	Traumatic/6	MCS-(2-3-2-2-0-2)	0/2	0/2	MCS-(2-3-2-2-1-2)	0/2	0/2

Notes: ^1^ CRS-R includes 6 sub-scales: Auditory Function Scale, Visual Function Scale, Motor Function Scale, Oromotor/Verbal Function Scale, Communication Scale, Arousal Scale

^2^ Functional Object Use is included in Motor Function Scale, and patients will be diagnosed as EMCS if get score in this item

This study has been designed in line with the CONSORT recommendations for reporting randomized trials (see Additional file 1).

Patients using a central nervous system stimulant, neuro-muscular blocking agents, or sedative for 24 h, with unstable status, concurrent disease (e.g., pyrexia, pneumonia, diarrhea), or dyskinesia of the upper limbs (i.e., motor sub-scale scores < 1 by using noxious stimuli to upper limbs), or receiving hyperbaric oxygen treatments within 2 h were excluded.

CRS-R assessment tools suggested by the CRS-R (cup and comb) and personalized stimulants (cigarette, paper, pen, mobile phone, tooth brush, fan, and lipstick) were used as assessment instruments. All personalized objects used were collected from patients’ bedsides in the hospital, or from their previous daily life.

Each patient was assessed at least twice by assessor “A” on different days within 1 week, with the CRS-R including the sub-item Functional Object Use as suggested by the CRS-R protocol. Patients were tested 2 more times using the CRS-R including Functional Object Use with personalized objects by a second experienced assessor “B” on the same day as by assessor “A”. The order of all the assessments was randomized and the time elapse between standard and ‘personalized’ assessments was set as short as possible (within 2 h). Personalized objects were chosen by family members, or nurses in cases where the patient lacked frequent company of family members. Objects were those be highly appreciated and/or used by patients in their previous daily live. Functional Object Use was evaluated by assessor “A” through standardized methodology as described in the CRS-R. In brief, the assessor placed one of the objects in the patient’s hand and instructed the patient that “Show me how to use a [name object].” Then, she placed the second object in the patient’s hand and restated the same instruction (see Additional file 2). All the objects were presented in a random order [2, 4].

During the assessment, patients were all subject to a standardized arousal facilitation protocol (i.e., deep pressure stimulation from the facial musculature to the toes was employed if needed, in order to prolong the length of time the patients maintained aroused, and this protocol was re-administered if patients showed sustained eye closure again or behavioral responsiveness ceased despite sustained eye opening [2, 4].)

Differences between Functional Object Use assessed by non-personalized and personalized stimulants were analyzed using the McNemar test. Results were considered significant at *p* < 0.05.

Six months after completion of the protocol, family members and/or nursing staff were asked to perform the CRS-R including the personalized objects used for each patient.

## Results

### Reaction to different objects

**Fig. 1:**
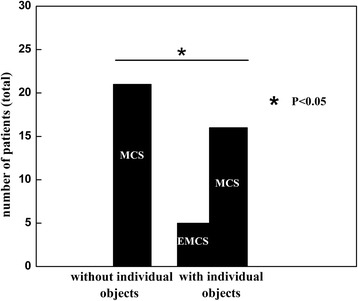
Functional Object Use with personalized objects. Number of patients diagnosed as MCS and EMCS as a function of the employed assessment (with/without personalized objects)

The incidence of Functional Object Use was significantly higher using personalized objects than non-personalized objects in the CRS-R (χ^2^ = 5, df = 1, p < 0.05, Fig. 1).] Five out of 21patients who were diagnosed as MCS (1 MCS+, 4 MCS-) without using personalized objects, were however re-diagnosed as EMCS with personalized objects, making a 23.8% diagnosis improvement (see details in Table 1). Four of the 5 patients (1 MCS+, 3 MCS-) only responded to personalized objects, but had no response to the non-personalized objects. The other patient previously diagnosed as MCS- was able to functionally use both one personalized object (mobile phone) and one non-personalized object (comb). Patients made an action of paper folding when investigators showed them a piece of paper (i.e., patient 1 and patient 3); they drew lines on the paper when given the pen (i.e., patient 1 and patient 3); they took the phone and shifted it closer to their ears like answering the phone when displayed a phone (i.e., patient 8 and patient 9); they moved the tooth brush around the mouth when given a tooth brush (i.e., patient 8); and they moved the comb to their heads when given a comb (i.e., patient 9).

Moreover, at 6 months follow-up all of the 5 EMCS patients were reportedly able to use the same personalized objects as tested before in a manner score-able as functional object use.

## Discussion

The aim of the study was to determine whether the assessment of Functional Object Use in DOC patients was influenced by the choice of objects. In this study, diagnosis of 5 patients changed when personalized objects were employed instead of the two the standard CRS-R protocol suggests.

Our results are in line with a large amount of research showing that the elicitation of patients’ behavioral response can be improved when their personal experience and personal preferences are taken into consideration. Several previous studies found that using the patient’s own name is more suitable to elicit a behavioral response as compared to a standard sound [14, 15]. Di et al.’s study proved that using a mirror (auto-referential stimuli) during the assessment of visual fixation in patients with DOC can elicit higher positive response rate compared to other neutral stimuli [16]. Furthermore, our results are in accordance with a recent study by Stenberg et al. [17], demonstrating improved behavioral responses with personalized stimuli in the visual sub-scale during the CRS-R assessment in 3 DOC patients. The emotional richness and complexity of personally relevant stimuli are considered to be crucial in promoting active responses of covert behaviors during assessment for DOC patients [7, 11, 12, 18].

Notably, we used a cigarette, paper, pen, mobile phone, tooth brush, fan and the standard comb and cup (as recommended in the CRS-R) and compared the frequency of behavioral response elicited by the different stimuli in 21 patients diagnosed as MCS. Patients for example made the action of origami shipshape when investigators showed the paper (i.e., patient 1 and patient 3); they drew lines on the paper when given the pen (i.e., patient 1 and patient 3); they took the phone and shift closer to their ears like answering the phone when displaying the phone (i.e., patient 8 and patient 9); they moved the tooth brush to around the mouth when given the tooth brush (i.e., patient 8); and they moved the comb to the head when given the comb (i.e., patient 9). Reaction of non-personalized objects included any movement that was not related to the item. The choice of objects was based on reports from family and clinicians from observations of affective behaviors like smiling, laughing, frowning, crying that occur spontaneously in response to the personal objects (cigarette, paper, pen, mobile phone, tooth brush, fan). One out of the five patients (i.e., patient 9) showed “Functional Object Use” to both a personalized object and a non-personalized object. When the non-personalized objects (comb and cup) were presented, the patient only showed a motor response to the comb and was diagnosed as MCS-. However, after being given personalized objects, he could use both the comb and a phone and was therefore re-diagnosed as EMCS. This indicates that personally relevant stimuli might specifically be important for those patients on the edge between MCS and EMCS.

In addition, patient 9 and patient 10 showed a parallel improvement on the motor and the arousal sub-scale while using personalized objects. This might be in line with the mood and arousal hypothesis put forward in previous studies [7]. Thus, the choice of personalized objects seems to be crucial to the accurate assessment of DOC patients’ conscious state [11, 18]. As the score of the motor sub-scale was stable across the repeated testing of the CRS-R, and the assessments were counterbalanced, it is unlikely that fluctuations in motricity underly differences in responses to personally relevant objects compared to neutral objects.

Outcome information from the family at 6 months after this study showed that all 5 patients were able to use the personalized objects (but not one or both of the neutral objects except for patient 9)) in a similar way as during the study. It indicates that patients were in a stable state and no behavioral improvement was expected to have occurred in the short period of the study. This is corroborated by the information that no improvement in conscious state was observed by the family or nursing staff (behaviors indicative of upper severe disability or higher [19]). However, main outcome measures were performed using interviews with families, and results should obviously be interpreted with care due to possible family bias.

## Conclusions

Personalized objects employed here seemed more sensitive to elicit patients’ responses as compared to common objects (e.g., comb, cup) during the assessment of Functional Object Use in patients with DOC.

## Additional files

Additional file 1: CONSORT 2010 checklist of information to include when reporting a randomised trial. (DOC 217 kb)

Additional file 2: The details of Function Object Use with personalized object. (DOCX 25 kb)

## Abbreviations

CRS-R: Coma Recovery Scale-Revised
DOC: Disorders of consciousness
EMCS: emergence from MCS
fMRI: Functional magnetic resonance imaging
MCS: Minimally conscious state
VS/UWS: Vegetative state/unresponsive wakefulness syndrome
