# Supplementary material for: Personalized objects can optimize the diagnosis of EMCS in the assessment of functional object use in the CRS-R: a double blind, randomized clinical trial
Source: BMC Neurol. 2018 Apr 12;18:38. doi: 10.1186/s12883-018-1040-5 (PMC5897931; doi:10.1186/s12883-018-1040-5)
Supplement: Supplementary file 2 — The details of Function Object Use with personalized object. (DOCX 25 kb) [file 12883_2018_1040_MOESM2_ESM.docx]

Additional file 2: The details of Function Object Use with personalized object

| Patient | Age (year)/Gender | Etiology/  Time since injury (month) | CRS-R^1^ | | | CRS-R Personalized objects | | |
| --- | --- | --- | --- | --- | --- | --- | --- | --- |
|  |  |  | Diagnosis | Functional Object Use^2^ | | Re-Diagnosis | Functional Object Use | |
|  |  |  |  | comb | cup |  | Object 1 | Object 2 |
| 1 | 41/M | Traumatic  /8 | MCS- (2-1-3-2-0-3) | 0/2 | 0/2 | EMCS (1-1-6-1-0-3) | Paper  (2/2) | Pen  (2/2) |
|  |  |  | MCS- (2-1-3-2-0-3) | 0/2 | 0/2 |  |  |  |
| 2 | 37/F | Non-  traumatic  /6 | MCS+ (2-1-2-2-1-2) | 0/2 | 0/2 | MCS+ (2-1-2-2-1-2) | 0/2 | 0/2 |
|  |  |  | MCS+ (2-1-2-2-1-2) | 0/2 | 0/2 |  |  |  |
| 3 | 62/M | Non-  traumatic  /3 | MCS+ (3-5-3-2-1-3) | 0/2 | 0/2 | EMCS (3-5-6-2-1-3) | Paper  (2/2) | Pen  (2/2) |
|  |  |  | MCS+ (3-5-3-2-1-3) | 0/2 | 0/2 |  |  |  |
| 4 | 44/F | Traumatic  /7 | MCS+ (2-1-2-2-1-2) | 0/2 | 0/2 | MCS+ (2-1-2-2-1-2) | 0/2 | 0/2 |
|  |  |  | MCS+ (2-1-2-2-1-2) | 0/2 | 0/2 |  |  |  |
| 5 | 62/M | Traumatic  /3 | MCS+ (1-3-3-2-0-2) | 0/2 | 0/2 | MCS+ (3-3-3-3-1-2) | 0/2 | 0/2 |
|  |  |  | MCS+ (3-3-3-3-1-2) | 0/2 | 0/2 |  |  |  |
| 6 | 67/M | Non-  traumatic  /5 | MCS+ (3-1-4-1-0-2) | 0/2 | 0/2 | MCS+ (3-1-4-1-0-2) | 0/2 | 0/2 |
|  |  |  | MCS+ (3-1-4-1-0-2) | 0/2 | 0/2 |  |  |  |
| 7 | 67/M | Traumatic  /10 | MCS- (0-2-2-1-0-2) | 0/2 | 0/2 | MCS- (0-2-2-1-0-2) | 0/2 | 0/2 |
|  |  |  | MCS- (0-2-2-1-0-2) | 0/2 | 0/2 |  |  |  |
| 8 | 62/M | Traumatic  /3 | MCS- (1-3-1-1-0-2) | 0/2 | 0/2 | EMCS (1-3-6-1-0-2) | Phone  (2/2) | Tooth Brush  (2/2) |
|  |  |  | MCS- (1-3-1-1-0-2) | 0/2 | 0/2 |  |  |  |
| 9 | 70/M | Non-  traumatic  /18 | MCS- (2-1-2-1-1-2) | 2/2 | 0/2 | EMCS (1-2-6-1-0-3) | Comb  (2/2) | Phone  (2/2) |
|  |  |  | MCS- (2-1-2-1-1-2) | 2/2 | 0/2 |  |  |  |
| 10 | 45/M | Non-  traumatic  /10 | MCS- (2-0-1-2-0-2) | 0/2 | 0/2 | EMCS (3-5-6-2-1-3) | Phone  (2/2) | Fan  (2/2) |
|  |  |  | MCS- (2-0-3-1-0-2) | 0/2 | 0/2 |  |  |  |
| 11 | 50/F | Non-  traumatic  /6 | MCS+ (3-5-1-2-1-2) | 0/2 | 0/2 | MCS+ (3-5-1-2-1-2) | 0/2 | 0/2 |
|  |  |  | MCS+ (3-5-1-2-1-2) | 0/2 | 0/2 |  |  |  |
| 12 | 58/F | Traumatic  /6 | MCS+ (2-3-2-2-1-2) | 0/2 | 0/2 | MCS+ (2-2-2-2-1-2) | 0/2 | 0/2 |
|  |  |  | MCS+ (2-2-2-2-1-2) | 0/2 | 0/2 |  |  |  |
| 13 | 58/M | Non-  traumatic  /3 | MCS+ (4-5-2-3-1-3) | 0/2 | 0/2 | MCS+ (4-5-2-3-1-3) | 0/2 | 0/2 |
|  |  |  | MCS+ (4-5-2-3-1-3) | 0/2 | 0/2 |  |  |  |
| 14 | 62/M | Traumatic  /3 | MCS+ (4-5-4-2-1-2) | 0/2 | 0/2 | MCS+ (4-5-4-2-1-2) | 0/2 | 0/2 |
|  |  |  | MCS+ (4-5-4-2-1-2) | 0/2 | 0/2 |  |  |  |
| 15 | 54/F | Non-  traumatic  /2 | MCS+(4-5-4-2-0-2) | 0/2 | 0/2 | MCS+(4-5-4-2-0-2) | 0/2 | 0/2 |
|  |  |  | MCS+(4-5-4-2-0-2) | 0/2 | 0/2 |  |  |  |
| 16 | 58/M | Non-  traumatic  /5 | MCS-(2-3-3-1-0-2) | 0/2 | 0/2 | MCS-(2-3-3-1-0-2) | 0/2 | 0/2 |
|  |  |  | MCS-(2-3-3-1-0-2) | 0/2 | 0/2 |  |  |  |
| 17 | 15/M | Traumatic  /9 | MCS-(2-3-2-1-0-2) | 0/2 | 0/2 | MCS-(2-3-2-1-0-2) | 0/2 | 0/2 |
|  |  |  | MCS-(2-3-2-1-0-2) | 0/2 | 0/2 |  |  |  |
| 18 | 61/F | Traumatic  /1 | MCS-(1-1-4-1-0-2) | 0/2 | 0/2 | MCS-(2-1-4-1-0-2) | 0/2 | 0/2 |
|  |  |  | MCS-(2-1-4-1-0-2) | 0/2 | 0/2 |  |  |  |
| 19 | 65/M | Non-  traumatic  /9 | MCS+(4-5-2-2-0-2) | 0/2 | 0/2 | MCS+(4-5-2-2-0-2) | 0/2 | 0/2 |
|  |  |  | MCS+(4-5-2-2-0-2) | 0/2 | 0/2 |  |  |  |
| 20 | 70/M | Traumatic  /4 | MCS-(2-3-3-1-0-2) | 0/2 | 0/2 | MCS-(2-3-3-1-0-2) | 0/2 | 0/2 |
|  |  |  | MCS-(2-3-3-1-0-2) | 0/2 | 0/2 |  |  |  |
| 21 | 40/M | Traumatic  /6 | MCS-(2-3-2-2-0-2) | 0/2 | 0/2 | MCS-(2-3-2-2-1-2) | 0/2 | 0/2 |
|  |  |  | MCS-(2-3-2-2-0-2) | 0/2 | 0/2 |  |  |  |

Notes:

1. CRS-R includes 6 sub-scales: Auditory Function Scale, Visual Function Scale, Motor Function Scale, Oromotor/Verbal Function Scale, Communication Scale, Arousal Scale.

2. Functional Object Use is included in Motor Function Scale, and patients will be diagnosed as EMCS if get score in this item.
